# Diphtheria

**Published:** 1905-09-09

**Authors:** 


### DIPHTHERIA.

In no single disease has there been greater practical progress than in the diagnosis and treatment of diphtheria. But with this progress, with recent developments in the direction of early recognition and more exact differential diagnosis we are brought face to face with difficulties that are entirely new and which demand still further advance and research for their solution. These difficulties were brought out by the discussion on "Diphtheria with especial reference to the infectivity and notification of the latent forms" introduced by Watson Williams at the Leicester meeting of the British Medical Association. It is universally recognised that diphtheria is a disease due to infection by the Klebs-Loeffler bacillus, and bacteriologists have enabled us to differentiate between true diphtheria and other conditions which clinically are indistinguishable from the true disease, except by bacteriological tests—*e.g.* the pseudo-membranous tonsillitis or pharyngitis due to Vincent's spirillum and the fusiform bacillus, or that which has occurred in connection with the bacillus coli communis, streptococci or staphylococci—while on the other hand it has proved to us that oftentimes the true diphtheria is unattended by any visible membrane, or indeed any false membrane at all, there being no local clinical manifestation of the infection beyond a tonsillitis or pharyngitis in no way differing in appearance from that due to cold, rheumatism, or any other simple inflammatory condition.

Thus it came to be believed that the bacteriologist had effectively banished all difficulties in the diagnosis of diphtheria, given an inflammatory condition of the fauces, of the nasal mucosa, of the larynx or trachea, or even a sore on the finger, the vulva or anus, associated with the Klebs-Loeffler bacillus, that was diphtheria. But a closer study of the Klebs-Loeffler bacillus revealed its extreme polymorphism, and now there are about twenty or more morphological varieties described, some irregular pear-shaped, some long and thin, others short and relatively thick, some with methyl blue staining at either end, some showing several round granular staining particles throughout the whole length of the bacillus, some with barred staining with clear spaces between, some staining evenly, solid staining. Then again, these stained bacilli are sometimes found to be non-virulent and perfectly innocuous when injected into rabbits, which are very susceptible to virulent diphtheria bacilli. Clearly a local inflammation associated with non-virulent bacilli, however closely they resembled in morphological characteristics the true virulent Klebs-Loeffler bacillus could not be regarded as clinical diphtheria, and as these diphtheroid bacilli were found in some conjunctival conditions, xerosis bacilli, and in pulmonary gangrene, in noma, in cancrum oris, it was evident that the bacteriologist required further tests before he could rely on simple staining of diphtheria-like bacilli to make a diagnosis. Then again we have to consider the relationship of some of the short solid forms which resemble, or are indistinguishable by the microscope from, the pseudo-diphtheria bacillus which is generally described as Hoffmann's bacillus.

Some bacteriological researches, such as those of Hewlett and Knight, appeared to show that Hoffmann's bacillus might, by growing it in certain media, be made to develop from the non-virulent Hoffmann type into the true and virulent Klebs-Loeffler type of bacillus. The same result was obtained by Salter, of the Jenner Institute, by inoculating finches with Hoffmann's bacilli, and he claimed in this way to have developed the true bacillus from the pseudo-bacillus. The results of these investigators appeared the more likely to be true, firstly, because it is certain that the virulence of the true bacillus varies in affected individuals, and that the Klebs-Loeffler bacillus in convalescents from diphtheria undergoes changes in type from the typical long granular staining form to the atypical short and less virulent form or to the involution forms, gradually losing some of its virulence from its growth on the mucosa of persons who have acquired immunity; and secondly, because the Hoffmann bacillus is very frequently found in the noses or throats of those patients who are convalescing. But the majority of bacteriologists do not accept Hewlett and Knight's and Salter's conclusions, and they regard the Hoffmann bacillus as a benign organism altogether distinct from the Klebs-Loeffler bacillus. That this must be the true view seems to be placed beyond doubt by the fact that Hoffmann's bacillus is found to exist freely in the throats and noses of a very large percentage of poor children; very often as many as 50 per cent. of groups of children who have not been affected by diphtheria, and who have not been in contact with diphtheria cases for several years, being affected with this organism. There are, however, many, well-known bacteriologists, who allow that the true Hoffmann bacillus is innocuous, but are unable to come to any decision when called upon to give an opinion on a culture from a suspected case, contenting themselves with stating that the culture yields organisms belonging to the diphtheroid group, but whether they are true or pseudo-diphtheria bacilli they cannot decide. The physician has then two courses open to him, he can decide from the clinical conditions that it is not diphtheria, or he can err on the side of safety and regard his patient as diphtheritic for the time being and await events. In the latter case he may send several culture-swabs—at intervals of a week—and the bacteriologist may continue to hedge and give no decision. We do not reflect on the bacteriologist; it is the only straightforward course to adopt until bacteriology has acquired more reliable methods in these difficult cases; but it shows that oftentimes we must regard bacteriological tests as untrustworthy, and that the clinician has to rely on clinical experience, or put his patients to great and often unnecessary expense and inconvenience, or perhaps lose his patients altogether, because they cannot understand, being in good health, that the discovery of a few germs of doubtful import is sufficient reason for being quarantined indefinitely.

We have drawn attention to the weak side of the culture test in the diagnosis of diphtheria, but it would be much easier to multiply examples of the enormous value that this science has proved itself to be in this same disease. Outbreaks of diphtheria are every now and then traced to some apparently healthy person who is the subject of diphtheritic rhinitis or diphtheritic nasal catarrh. The routine examination of all the noses and throats of an affected community may disclose a chronic cold in the nose of one of the healthy persons. There may be some pseudo-membrane in the nasal passages or there may be nothing visible but a sticky catarrhal secretion, and yet the culture may prove that the nasal passages are teeming with typical virulent diphtheria bacilli. The isolation of this individual until local treatment by antiseptics has completely got rid of all pathogenic organisms has time after time caused the total disappearance of all diphtheria in a group of persons, generally it is a school, although before the removal of the source of infection diphtheria had been cropping up from time to time notwithstanding the most rigid precautions.

